# Gastroenteritis is Less Severe But is More Often Associated With Systemic Inflammation in SARS-CoV-2-positive Than in SARS-CoV-2-Negative Children

**DOI:** 10.1097/INF.0000000000004001

**Published:** 2023-06-14

**Authors:** Gregorio P. Milani, Danilo Buonsenso, Paola Marchisio, Carlo Agostoni, Chiara Maria Corso, Alfredo Guarino, Marco Poeta, Francesco Proli, Alessandra Drosi, Rosa Morello, Andrea Lo Vecchio

**Affiliations:** From the * Pediatric Unit, Fondazione IRCCS Ca’ Granda Ospedale Maggiore Policlinico, Milan, Italy; † Department of Clinical Sciences and Community Health, Università Degli Studi di Milano, Milan, Italy; ‡ Department of Woman and Child Health and Public Health, Fondazione Policlinico Universitario A. Gemelli IRCCS, Rome, Italy; § Istituto di Igiene, Global Health Research Institute, Università Cattolica del Sacro Cuore, Rome, Italy; ¶ Department of Translational Medical Sciences, Section of Pediatrics. University of Naples Federico II, Naples, Italy.

## Abstract

This study aims to characterize the clinical and metabolic features of acute gastroenteritis in children with and without severe acute respiratory syndrome coronavirus 2 (SARS-CoV-2). A multicenter case–control study was conducted in 2022 including 200 children. Clinical data and laboratory tests were analyzed. Children with SARS-CoV-2 presented less frequently hyponatremia and metabolic acidosis, but more often systemic inflammation as compared with children without SARS-CoV-2.

The manifestations of severe acute respiratory syndrome coronavirus 2 (SARS-CoV-2) infection in children range widely. Unlike adults, children generally experience less severe acute infection with a lower fatality rate.^1,2^ However, severe clinical courses in childhood have been documented associated especially with conditions like long coronavirus disease (COVID) or multisystem inflammatory syndrome.^3,4^ Approximately one-third of children with COVID-19 present gastrointestinal symptoms, including vomiting and diarrhea. SARS-CoV-2 has been found to induce these symptoms through several mechanisms,^5^ but there is no detailed comparison available between acute gastroenteritis (AGE) associated with SARS-CoV-2 and with other enteric pathogens. Thus, this study aims to characterize the metabolic profile and severity of AGE in children with SARS-CoV-2 or other infectious causes.

## METHODS

We conducted a multicenter, case–control, retrospective study (January to December 2022) at the pediatric units of the Ca’ Granda Ospedale Maggiore Policlinico, Milan, Fondazione Policlinico Universitario “A. Gemelli,” Rome and at the Department of Translational Medical Sciences of the University of Naples “Federico II,” Naples (Italy). Children hospitalized for AGE, defined as a reduction in the consistency of stools and/or an increase (≥3/d) in frequency of evacuations^6^ and underwent a standard nasopharyngeal swab^7^ for SARS-CoV-2 molecular test at admission were eligible. Children with AGE who tested positive for SARS-CoV-2 were defined as cases. For each case, an age-matched (±6 months) child hospitalized for AGE who tested negative for SARS-CoV-2 infection was selected as a control. Children with respiratory diseases (eg, pneumonia), underlying conditions that could alter electrolytes or acid-base balance (eg, children with diabetes), or those without blood testing were excluded from the study. Cases of Multisystem Inflammatory Syndrome in Children were also ruled out. Data on age, sex, presence of fever (>37.9°C), history of vaccination against SARS-CoV-2, capillary refill time (≤2 vs. >2 seconds), need of intravenous rehydration, length of hospitalization, were extracted. Additionally, results of a venous blood sampling on admission, which included measurements of white blood cell count, C-reactive protein (CRP), urea, ionized sodium (measured by direct potentiometry^8^), potassium and chloride, pH, carbon dioxide pressure, bicarbonate, lactate and glycemia, were also obtained.

Stool samples were collected and analyzed for culture (Shigella, Salmonella and Campylobacter), Rotaviru*s* and Adenovirus fecal antigens and/or multiplex polymerase chain reaction (PCR) for enteropathogens among viruses, bacteria and parasites (FILMARRAY GI Panel – Biomerieux, Italy) according to single center protocols. The findings of these tests were recorded, when available.

All data were collected from the electronic medical records of the hospitals and included in a predefined database. CRP was defined as elevated when >1 mg/dL. Hyponatremia was defined as a circulating sodium level <135 mmol/L. Metabolic acidosis was diagnosed in cases with a pH ≤7.40 and circulating bicarbonate levels <20 mmol/L.^9^

Continuous data are reported as median and interquartile range (IQR), while categorical data are presented as frequency and percentage. For inferential statistics, the characteristics of children with and without SARS-CoV-2 were compared. Subsequently, the analyses were focused on children who had available microbiologic stool test results and were divided into the following groups: (a) children who tested positive for SARS-CoV-2 in the nasopharyngeal swab but negative for pathogenic enteric organisms from stool tests, (b) children who tested positive for both SARS-CoV-2 via nasopharyngeal swab and at least 1 pathogenic enteric organism from stool tests and (c) children who tested negative for SARS-CoV-2 in the nasopharyngeal swab but positive for at least 1 pathogenic enteric organism from stool tests. Children testing negative both for SARS-CoV-2 in the nasopharyngeal swab and for pathogenic enteric organism from the stools were not considered in the second step of the analyses. Mann-Whitney *U* test or Kruskal Wallis test were used to compare continuous variables whereas Fisher or χ^2^-test for categorical data, as appropriate. A *P* < 0.05 was considered as significant. Statistics was performed using R (version 3.5.3). The study was approved by the Ethic committee of Gemelli University Hospital (ID 3078).

## RESULTS

In the study period, we included 100 consecutive children (median age 1.8, IQR 0.3–5.5, years, 50 females) hospitalized for AGE and testing positive for SARS-CoV-2 in the nasopharyngeal swab. The patient’s enrollment flowchart is reported in the Figure, Supplemental Digital Content 1, http://links.lww.com/INF/F126. Other 100 children [median age 2.4 (0.9–5.4) years, 46 females] hospitalized for AGE and testing negative for SARS-CoV-2 were included as controls. Demographic characteristics and the proportion of children presenting with a capillary refill time >2 seconds were similar between the 2 groups (Table 1). The occurrence of fever was slightly more frequent in children testing positive for SARS-CoV-2 compared with those testing negative (N = 57, 57% vs. N = 42, 48%, respectively, *P* = 0.047) as well as the length of hospitalization was slightly higher in former as compared with latter group (median 3, IQR 2–5, vs. 1, IQR 1–4, days, *P* < 0.001). On contrary, intravenous rehydration was less common in children testing positive than in those testing negative for SARS-CoV-2 (56% vs. 93%, *P* < 0.001). Children with SARS-CoV-2 showed increased systemic inflammation compared with those testing negative, as demonstrated by median values of CRP [1.90 mg/dL (IQR 0.50–6.88) vs. 0.38 mg/dL (IQR 0.05–2.92), *P* < 0.001]. Accordingly, elevated CRP was more frequent among children with as compared with those without SARS-CoV-2 (63% vs. 36%, *P* = 0.0002). Hyponatremia (27% vs. 55%, *P* < 0.001) and metabolic acidosis (51% vs. 76%, *P* < 0.001) were less frequent in children with compared with those without SARS-CoV-2 (Table, Supplemental Digital Content 2, http://links.lww.com/INF/F127).

**TABLE 1.** Characteristics of the 200 Included Children Hospitalized for an Acute Gastroenteritis

	All	SARS-CoV-2 POS	SARS-CoV-2 NEG	*P* Value
N	200	100	100	
Age (y)	2.0 [0.5–5.4]	1.8 [0.3–5.5]	2.4 [0.9–5.4]	0.064
Males	104 (52)	50 (50)	54 (54)	0.671
Fever (>37.9°C)	99 (50)	57 (57)	42 (42)	**0.047**
Vaccination against SARS-CoV-2	4 (2.0)	0 (0)	4 (8.0)	0.121
Capillary refill time >2 s (yes)	26 (13)	16 (16)	8 (7.7)	0.082
Intravenous rehydration (yes)*	149 (75)	56 (56)	93 (93)	**<0.001**
Lenght of hospitalization, d	3 [1–4]	3 [2–5]	1 [1–4]	**<0.001**
Plasma level				
White blood cell count	10.12 [7.95–14.67]	8.81 [6.76–11.99]	10.57 [8.70–13.90]	**0.003**
Reactive-C Protein (mg/dL)	0.90 [0.16–4.28]	1.90 [0.50–6.88]	0.38 [0.05–2.92]	**<0.001**
Urea (mg/dL)	22 [13–30]	19 [12–28]	23 [14–34]	**0.01**
Whole blood level				
Sodium (mmol/L)	135 [132–137]	137 [134–138]	134 [131–136]	**<0.001**
Potassium (mmol/L)	4.4 [3.9–4.9]	4.5 [4.1–4.9]	4.2 [3.8–4.7]	**0.005**
Chloride (mmol/L)	103 [99–105]	102 [100–104]	103 [99–105]	0.699
pH	7.37 [7.34–7.42]	7.39 [7.34–7.45]	7.37 [7.33–7.40]	0.126
Carbon dioxide pressure (mm Hg)	32.3 [28.0–38.2]	36.5 [30.8–39.8]	31.0 [27.2–38.0]	**0.03**
Bicarbonate (mmol/L)	19.9 [16.6–23.3]	22.3 [19.5–24.2]	18.6 [16.0–22.8]	**0.001**
Lactate (mmol/dL)	1.71 [1.20–2.66]	2.3 [1.4–3.2]	1.65 [1.18–2.30]	**0.028**
Glucose (mg/dL)	88 [76–102]	90 [78–99]	87 [73–104]	0.462

* Limiting the comparison to patients receiving intravenous rehydration, no difference (4 [4–6]–4 [2–5] d, *P* = 0.06) was observed between the 2 groups.

SARS-CoV-2 indicates severe acute respiratory syndrome coronavirus 2.

A total of 137 (69%) of the 200 enrolled children underwent fecal microbiological tests. In most cases, a viral agent was found (adenovirus 20%, norovirus 20% and rotavirus 17%; Table, Supplemental Digital Content 3, http://links.lww.com/INF/F128). Twenty-five children tested negative for both SARS-CoV-2 in the nasopharyngeal swab and fecal enteropathogens.

Table, Supplemental Digital Content 4, http://links.lww.com/INF/F129, reports the characteristics of children with positive SARS-CoV-2 in the nasopharyngeal swab, and testing negative for other enteropathogens (N = 65), compared with those testing positive for both SARS-CoV-2 in the nasopharyngeal swab and at least 1 enteric pathogenic on the stools (N = 14), or SARS-CoV-2–negative children positive for at least 1 enteric pathogenic on the stools (N = 33). No difference for age, sex, occurrence of fever, time of capillary refill and length of hospitalization was observed among the 3 groups. The median CRP resulted significantly higher in children with SARS-CoV-2 with or without an enteric coinfection compared with SARS-CoV-2–negative children (*P* = 0.001). The number of children with elevated CRP was not different for the 3 groups (63%, 79% and 48%, respectively, *P* = 0.154, Table, Supplemental Digital Content 5, http://links.lww.com/INF/F130). Hyponatremia was less frequent in children testing positive only for SARS-CoV-2 as compared with the other 2 groups (17%, 36% and 55%, respectively, *P* < 0.001). A similar figure was observed for the presence of metabolic acidosis among the 3 groups (18%, 50% and 55%, respectively, *P* < 0.005).

## DISCUSSION

This study provides evidence that children with SARS-CoV-2 infection exhibit less severe metabolic changes compared with those with AGE caused by other pathogens. Specifically, the frequency of hyponatremia and metabolic acidosis in SARS-CoV-2 patients was lower than in the control group. Additionally, bicarbonate levels were mostly within normal ranges in children with SARS-CoV-2 infection, whereas they were significantly reduced in those with AGE caused by other etiologies. This indicates that children with AGE caused by other etiologies more frequently show a moderate-to-severe dehydration, as expected being this AGE caused by traditional infectious agents the main cause for hospital admission in European children.^10^

Children with SARS-CoV-2 infection had a longer hospital stay compared with the control group, but this difference was not significant when stratifying the children into 3 groups based on the presence of SARS-CoV-2 and other enteric pathogens. Nonetheless, a prolonged hospitalization is not necessarily indicative of the severity of AGE. It is possible that the need for stringent isolation measures and specific infection control protocols may have prolonged the hospital stay beyond what was clinically necessary, at least in some cases. This hypothesis is further supported by the observation that analyzing only children who received intravenous fluids, the length of hospital stay was similar between children with and without SARS-CoV-2.

We observed a significant increase in inflammatory markers among children with SARS-CoV-2 infection compared with those admitted for AGE caused by other enteropathogens. It is known that in children with AGE, the level of CRP is not able to accurately discriminate between different infectious etiologies, and a normal value does not exclude the possibility of bacterial gastroenteritis.^10^ The increase of CRP in patients with SARS-CoV-2 is likely to indicate systemic involvement during this infection. Furthermore, previous studies have shown that children hospitalized for COVID-19 with diarrhea have increased levels of inflammatory markers compared with those without gastrointestinal involvement.^11^ On the other hand, it should be noted that median levels of CRP were not markedly elevated both in patients with and without SARS-CoV-2.

The retrospective nature and lack of follow-up after discharge are the major limitations of the present study. Relevant information such as the number of bowel movements could not be collected. Additionally, microbiological investigations were not performed in all children and depended on the procedures of each canter. Information on immunization against rotavirus was not available. Although no SARS-CoV-2 infections were observed in the vaccinated children, this outcome did not reach statistical significance. We hypothesize that the absence of a significant difference may be attributed to the predominance of subjects <5 years of age in our sample, considering that the vaccination approval for this age group was not approved in Italy for the majority of 2022. Finally, we did not have data on SARS-CoV-2 strains associated to AGE. However, the study also has strengths. It included age-matched children from different hospitals and electrolytes, particularly sodium, were measured by direct potentiometry as recommended by international authorities.^12,13^

In conclusion, these data point out that SARS-CoV-2 is associated with a lower risk of dehydration, hyponatremia and metabolic acidosis, but higher frequency of systemic inflammation compared with other pathogens. Further prospective studies to characterize AGE in children with and without SARS-CoV-2 are needed.

## Supplementary Material


