# Supplementary material for: Gastroenteritis is Less Severe But is More Often Associated With Systemic Inflammation in SARS-CoV-2-positive Than in SARS-CoV-2-Negative Children
Source: Pediatr Infect Dis J. 2023 Jun 14;42(9):e320–2. doi: 10.1097/INF.0000000000004001 (PMC10417221; doi:10.1097/INF.0000000000004001)
Supplement: Supplementary file 1 [file inf-42-e320-s001.docx]

**Supplementary Digital Content 1.** Flowchart of patient’s enrollment
