# Supplementary material for: Gastroenteritis is Less Severe But is More Often Associated With Systemic Inflammation in SARS-CoV-2-positive Than in SARS-CoV-2-Negative Children
Source: Pediatr Infect Dis J. 2023 Jun 14;42(9):e320–2. doi: 10.1097/INF.0000000000004001 (PMC10417221; doi:10.1097/INF.0000000000004001)
Supplement: Supplementary file 2 [file inf-42-e320-s002.docx]

**Supplemental Digital Content 2.** Frequency of elevated CRP (>1 mg/dL), hyponatremia (circulating sodium <135 mmol/L) and metabolic acidosis (pH ≤7.40 and circulating bicarbonate levels <20 mmol/L) among the 100 cases (SARS-CoV-2 positive) and 100 controls (SARS-CoV-2 negative) with acute gastroenteritis. Data are given as frequency and (percentage)

|  | **SARS-CoV-2 POS** | **SARS-CoV-2 NEG** | **P-value** |
| --- | --- | --- | --- |
| Elevated CRP | 62 (63) | 36 (36) | **<0.001** |
| Hyponatremia | 26 (27) | 55 (55) | **<0.001** |
| Metabolic Acidosis | 45 (51) | 32 (76) | **0.005** |

There were 2 missing data for PCR, 3 for sodium and 69 for acid-base balance values.
