# Supplementary material for: Gastroenteritis is Less Severe But is More Often Associated With Systemic Inflammation in SARS-CoV-2-positive Than in SARS-CoV-2-Negative Children
Source: Pediatr Infect Dis J. 2023 Jun 14;42(9):e320–2. doi: 10.1097/INF.0000000000004001 (PMC10417221; doi:10.1097/INF.0000000000004001)
Supplement: Supplementary file 3 [file inf-42-e320-s003.docx]

**Supplemental Digital Content 3.** Pathogenic enteric organisms other than SARS-CoV-2 detected from the stools of 46 children hospitalized for acute gastroenteritis. In eight subjects, one or more pathogen was detected.

| **Micro-organism** | **N** | **%** |
| --- | --- | --- |
| Adenovirus | 12 | 20 |
| Norovirus | 12 | 20 |
| Rotavirus | 10 | 17 |
| *Salmonella* | 6 | 10 |
| Astrovirus | 4 | 6.8 |
| *Campylobacter Jejuni* | 3 | 5.1 |
| *Clostridium difficilis* | 3 | 5.1 |
| *Blastocystis hominis* | 2 | 3.4 |
| *Escherichia Coli* | 4 | 6.8 |
| Sapovirus | 2 | 3.4 |
| Cytomegalovirus | 1 | 1.7 |
