# Supplementary material for: Gastroenteritis is Less Severe But is More Often Associated With Systemic Inflammation in SARS-CoV-2-positive Than in SARS-CoV-2-Negative Children
Source: Pediatr Infect Dis J. 2023 Jun 14;42(9):e320–2. doi: 10.1097/INF.0000000000004001 (PMC10417221; doi:10.1097/INF.0000000000004001)
Supplement: Supplementary file 4 [file inf-42-e320-s004.docx]

**Supplemental Digital Content 4**. Characteristics of the 112 children testing positive for SARS-CoV-2 and/or for at least one pathogenic enteric organism from the stool testing.

|  | **SARS-CoV-2 positive (pathogenic enteric organism negative)** | **SARS-CoV-2 plus pathogenic enteric organism positive** | **Pathogenic enteric organism positive (SARS-CoV-2 negative)** | **P-value** |
| --- | --- | --- | --- | --- |
| N | 65 | 14 | 33 |  |
| Age, years | 1.9 [0.2-5.9] | 2.7 [0.7-3.5] | 1.6 [0.7-5.1] | 0.706 |
| Males | 32 (49) | 6 (43) | 18 (55) | 0.738 |
| Fever (>37.9°C) | 37 (57) | 9 (64) | 16 (48) | 0.606 |
| Vaccination against SARS-CoV-2 | 0 (0) | 0 (0) | 1 (0.3) | 0.420 |
| Capillary refill time >2 sec. (yes) | 10 (15) | 3 (4.6) | 4 (7.1) | 0.195 |
| Intravenous rehydration (yes) | 25 (38) | 11 (79) | 32 (97) | **<0.0001** |
| Length of hospitalization, days | 4 [2-5] | 5 [4-6] | 4 [2-5] | 0.076 |
| Plasma level |  |  |  |  |
| White blood cell | 8.64 [6.99-11.27] | 9.48 [7.15-16.33] | 11.17 [8.80-13.93] | **0.011** |
| Reactive-C Protein (mg/dL) | 1.60 [0.50-8.40] | 2.86 [1.08-11.38] | 0.69 [0.50-2.93] | **0.001** |
| Urea, mg/dL | 21 [14-28] | 15 [8-24] | 17 [12-31] | 0.39 |
| Whole blood level |  |  |  |  |
| Sodium, mmol/L | 137 [135-139] | 136 [131-137] | 134 [131-137] | **<0.0001** |
| Potassium, mmol/L | 4.7 [4.3-4.5] | 4.2 [4.0-4.5] | 4.2 [3.7-4.6] | **<0.0001** |
| Chloride, mmol/L | 104 [101-105] | 103 [101-105] | 103 [99-106] | 0.972 |
| pH | 7.39 [7.35-7.47] | 7.37 [7.34-7.39] | 7.35 [7.32-7.40] | 0.113 |
| Carbon dioxide pressure (mm Hg) | 35.0 [30.5-42.8] | 34.0 [30.0-38.0] | 31.0 [29.0-38.0] | 0.609 |
| Bicarbonate (mmol/L) | 23.2 [20.3-24.2] | 19.7 [16.2-20.7] | 18.6 [16.0-22.8] | **0.022** |
| Lactate, mmol/dL | 2.15 [1.50-2.98] | 2.40 [2.30-3.00] | 1.40 [1.05-2.60] | 0.126 |
| Glucose, mg/dL | 88 [79-97] | 87 [74-101] | 87 [74-102] | 0.944 |
