# Supplementary material for: Gastroenteritis is Less Severe But is More Often Associated With Systemic Inflammation in SARS-CoV-2-positive Than in SARS-CoV-2-Negative Children
Source: Pediatr Infect Dis J. 2023 Jun 14;42(9):e320–2. doi: 10.1097/INF.0000000000004001 (PMC10417221; doi:10.1097/INF.0000000000004001)
Supplement: Supplementary file 5 [file inf-42-e320-s005.docx]

**Supplemental Digital Content 5.** Frequency of elevated CRP (>1 mg/dL), hyponatremia (circulating sodium <135 mmol/L) and metabolic acidosis (pH ≤7.40 and circulating bicarbonate levels <20 mmol/L) in the following three groups: children testing positive for SARS-CoV-2 but negative for pathogenic enteric organisms from the stools (N=65), children testing positive both for SARS-CoV-2 and for at least one pathogenic enteric organism from the stools (N=14), and children testing negative for SARS-CoV-2, but positive for at least one pathogenic enteric organism from the stools (N=33). Data are given as frequency and (percentage)

|  | **SARS-CoV-2 POS alone** | **SARS-CoV-2 POS plus another micro-organism** | **SARS-CoV-2 negative / another micro-organism** | **P-value** |
| --- | --- | --- | --- | --- |
| Elevated CRP | 41 (63) | 11 (79) | 16 (48) | 0.154 |
| Hyponatremia | 11 (17) | 5 (36) | 18 (55) | **<0.001** |
| Metabolic acidosis | 3 (18) | 2 (50) | 18 (55) | **0.002** |

There were 58 missing data for acid-base balance values
